# Hydrotherapy in the Home

**Published:** 1913-10

**Authors:** J. H. Neal

**Affiliations:** Atlanta, Ga.


### HYDROTHERAPY IN THE HOME.

J. H. Neal, M. D., Atlanta, Ga.

---

In these days when we hear so much about the terrible dangers of resorting to patent medicines, exploited in the daily papers as being able to combat every phase of disease from a cold in the head to the worst form of tertiary syphilis, we are apt to forget that some household remedies that we know to be harmful, are looked upon as so many lares et penates that the housekeeper would not feel safe without. The iconoclast, in destroying these idols, falls short in not giving something to take their place.

There are many poor people with whom we are all acquainted that in hope that they may find a short cut to health when lost, fall easy victims to the unscrupulous advertiser with his vaunted remedy for all the ills of humanity. Instead of calling the physician, the patient may already have in mind the very remedy that he may think will be just the thing to try in this case.

A headache may call into use the so-called harmless headache powder, or sleeplessness be the trap that will catch the unwary in a habit that will bind its victim in chains as strong as iron.

Your essayist wishes to show that a great many indications may be met in a more safe and sane way by nature's almost universal remedy.

Let us, however, lay down a few rules that one needs to bear in mind.

Don't go by the rule of thumb, guessing at the temperature of the person or of the water that is used.

Have a clinical thermometer and a water thermometer so as to know what temperature you are using. It will take very little time and trouble to teach a mother how to read and to a certain extent interpret what she reads.

Much distress of mind could be avoided if in every family the mother with children under her care, could have a clinical thermometer and be taught the use of it in the ordinary disturbances that come to childhood.

Many a child has returned from school after a hard day of study and play with a headache and a high temperature from an acute indigestion brought on by his too strenuous activities after eating and neglect of the calls of nature, to be dosed by the fond parent with some patent household remedy, when all that was necessary was an inforced abstinence from food, a warm bath, and rest in bed. Again, a child may partake of indigestible articles, such as green fruit, cheese, pickles, and candies, to be dosed with the deadly "pain-killer" to his serious injury. Much less serious would have been the damage had the mother or nurse resorted to a more rational method of treatment by water. An enema by means of the fountain syringe, fomentations to the painful part, followed by a simple laxative would have well met the indications.

A child is burned seriously through the clothing taking fire at the open grate. There is no dressing equal to the continuous warm bath, temperature from 95 to 99 degrees Fahrenheit.

Dr. A. Rose, of New York City, writing of the continuous warm bath says, "The action of the continuous bath in burns is manifold. It gives almost immediate, and even complete, relief from pain, and can be regarded as the most excellent anodyne. Even if it offered no other advantage, it would be of great value on account of this soothing effect when the pains are most excruciating. Another advantage of the warm-water treatment is that the water penetrates the burnt tissues, in consequence of which they remain moist and soft. Without the immersion the cuticle which has been destroyed in its whole depth would harden and form an impenetrable covering over the underlying parts. Immersed in water, tissues that have become gangrenous cannot dry up, but remain moist. They detach themselves easily and are washed away after having become detached. Thus the wound is constantly kept clean. There is no accumulation of pus, there are no crusts of desiccated wound secretions, and, most essential no dressing is required."

"When reading history or studying ethnography we feel horrified upon learning of barbarous cruelties, but nothing can surpass medical cruelty when cases of extensive burns are treated with all kinds of dressings instead of resorting to the most rational, the ideal method of placing the patient into a warm bath. I happened to see a patient with extensive burns in excruciating agony, facies hippocratica, in one of our public hospitals, whose wounds were being dressed by two nurses. I spoke to the physicians in attendance, suggesting the bath to relieve at least the frightful sufferings. They had never heard of such treatment and refused to follow my suggestion. There are certainly many cases which without the advantage of this means would prove fatal, while with the aid of the continuous warm bath they make a good recovery."

Nor is the application of the remedy a very hard matter, as this is done by arranging a sheet so as to form a hammock in the tub in which the child lies, covered by the water at the temperature 95 to 99 degree Fahrenheit. The water should be changed two or three times a day, a wet sheet being laid over the patient while the water is being renewed.

Who that has watched the trend of events in modern medical methods has not seen that the greatest advances have been made along the path of preventive medicine rather than of curing after the disease has become seated in the individual.

Here is a broad field for hydrotherapy in the home. As a prophylactic for child or adult a daily bath taken in the morning before dressing would not only prevent skin troubles, but would do much towards guarding the individual from the effects of sudden changes with their resulting colds.

A shower bath in the home is a desideratum. Even to one of moderate means it is a thing that can be attained without great outlay. To one accustomed to such a bath it becomes a hardship to be deprived of it. Where there is no water piped into the house an ordinary five gallon tin can with a little ingenuity can be made to render good service. By attaching the nozzle of a watering pot with fine perforations to the above mentioned can and placing it at the elevation desired over an ordinary wash tub a good shower may be had.

There is no tonic measure more exhilarating than the rough mitten friction applied in the morning before arising from the bed. The worst obstacle to this is that is requires the services of an attendant. All the other requirements can be readily met. A basin of water with a small piece of ice and a rough mitten with towels to protect the bed clothing are all that are necesary. The mitten may be made of coarse toweling or rough mohair may be utilized for the purpose. As a treatment in pneumonia the writer has found it a good adjunct to other treatment. When we consider that nearly one-third of the blood in the whole body can be held in the skin, one can readily see how internal congestion can be relieved in a rational and harmless way.

Colds and grippe can be handled in the home with good results by placing the patient in a hot leg bath in warm blankets so that free perspiration will be induced. By having him drink freely of hot lemonade and wrapping him in the bed coverings a gentle perspiration may be prolonged. This should be followed in the morning with the above mentioned cold mitten friction to prevent the taking of more cold on arising from the bed.

Hot blanket packs and wet packs can be managed in the home to good effect.

Constipation can be combatted by means of the graduated enema. Starting with a large coloclyster of warm water the first morning after breakfast and each day following, decreasing the amount of water and lowering the temperature until a very small amount of cold water will be sufficient to ensure a good action from the bowels. If the method is followed with proper regularity, by the time one has completed the first course the habit will have been established with no further need of water.

The rubber hot water bottle is an article quite indispensable in all well regulated families. Many pains can be relieved and much comfort given a patient by a means so simple. Moist heat is usually more effectual in relieving pain than dry heat. To obtain moist heat from the hot water bottle, one places around it a moist cloth over it a dry one to come next to the skin. In this way a very good fomentation can be given.

To a sleepless patient the neutral bath (temp. 95 to 97) will bring relief where the patient is not suffering actual pain. There is no therapeutic measure more quieting.

The hot and cold trunk pack meets the indication in the vomiting of pregnancy as no other means will. This was the method introduced by the eminent hydrotherapist Winternitz of Vienna. The method is this: a wet girdle is placed around the patient covering the body from the breasts to the pubis. Over this is placed a heavy dry flannel. At the same time the hot water bottle is laid over the stomach under the flannel. This is to be continuous or repeated as necessary. With regulation of the diet, the above has rescued some that were given up to be operated upon to empty the uterus in order to control nausea.

Another most valuable therapeutic procedure is the application of hot and cold to the spine by means of the long spine bag and a piece of ice. This procedure is carried out by filling the spine bag with hot water and applying it to the spine for three to four minutes and then apply the ice to the same part by rubbing it two or three times up and down the spine. Repeat this three time, ending always with ice. The effect is most smoothing to a restless patient.

Would there were more of our professional men who understood the technique of the water treatments and could make use of them to save life.

136 Gordon St., Atlanta, Ga.

